# Testicular Photohyperthermia Mediated by Magnetic Nanoparticles: Implications for Male Fertility Control

**DOI:** 10.3390/molecules31071064

**Published:** 2026-03-24

**Authors:** Vanessa N. Lima, Juliana Lis M. Brito, Ana Bárbara R. Silva, Aline R. M. Marangon, Felipe M. Pimentel, Breno C. P. Coelho, Marcelo H. Sousa, Rodrigo B. Nunes, Paulo Eduardo N. Souza, Raquel Pazos, Sergio E. Moya, Carolina M. Lucci

**Affiliations:** 1 Instituto de Ciências Biológicas, Universidade de Brasília, Asa Norte, Brasilia 70910-900, DF, Brazil; vanessanicolaudelima@gmail.com (V.N.L.); julianalis.brito@gmail.com (J.L.M.B.); anabarbararocha@unb.br (A.B.R.S.); 2 Independent Researcher, Taguatinga 72015-015, DF, Brazil; imagepet.exames@gmail.com; 3 Independent Researcher, Brasilia 70684-200, DF, Brazil; felipemcmanus@gmail.com; 4 Instituto Federal de Brasília, Asa Norte, Brasilia 70830-450, DF, Brazil; breno.coelho@ifb.edu.br; 5 Faculdade de Ciências e Tecnologias em Saúde, Campus Universitário Ceilândia Sul, Universidade de Brasília, Ceilândia 72220-275, DF, Brazil; mhsousa@unb.br; 6 Instituto de Física, Universidade de Brasília, Asa Norte, Brasilia 70910-900, DF, Brazil; rodrigobsnunes@gmail.com (R.B.N.); psouza@unb.br (P.E.N.S.); 7 Centro de Investigación Cooperativa en Biomateriales (CIC biomaGUNE), Basque Research and Technology Alliance (BRTA), 20014 San Sebastian, Spain; rpazos@cicbiomagune.es (R.P.); smoya@cicbiomagune.es (S.E.M.)

**Keywords:** nanocastration, nanoparticle-mediated hyperthermia, disrupted fertility, seminiferous tubule degeneration, nanoparticle biodistribution

## Abstract

In search of a non-surgical alternative for male animal sterilization, this study investigated the use of gold-coated maghemite nanoparticles (γ-Fe_2_O_3_@Au) functionalized with citrate to produce testicular photohyperthermia (PHT). Wistar rats received an intratesticular injection of the fluid containing the nanoparticles (150 µL/testicle) followed by testicular irradiation with an LED light (808 nm). Testicular temperature was maintained at ~45 °C for 15 min. The results demonstrated a significant reduction in testicular volume and weight and sperm motility and normal morphology in PHT-treated animals, together with histopathological degeneration of seminiferous tubules. No treatment-related side effects or signs of systemic toxicity were observed. The biodistribution of the gold (Au) and iron (Fe) from the nanoparticles showed that the testes were the primary site of nanoparticle accumulation until day 56 post-treatment with possible renal excretion of Au. These findings support the prospect of testicular PHT mediated by γ-Fe_2_O_3_@Au nanoparticles as a neutering method for male animals.

## 1. Introduction

The growing concern over the increasing number of stray animals has driven the search for effective and ethical population control methods. Conventional surgical castration, although definitive, faces practical challenges that limit its large-scale application [1,2]. An ideal method for non-housed animals must be non-invasive, simple to perform, and definitive and effective after a single application and must not cause serious side effects.

Numerous non-surgical neutering strategies for male animals have been investigated, including chemical and physical methods. Chemical methods involve intratesticular injection of sclerosing agents, such as zinc gluconate and calcium chloride [2,3]. Although extremely simple to perform and commercially available for some time, chemical methods have never been widely accepted or used due to their side effects (pain, local inflammation, necrosis and ulceration) [2,4,5,6,7]. Physical methods take advantage of the sensitivity of spermatogenesis to high temperatures and have invested in ways to produce testicular heating using water baths, microwaves, or ultrasound [8,9,10,11,12]. These approaches were not successful because the effect was usually temporary or required multiple applications over several days or weeks to become definitive, making them impractical.

More recently, nanotechnology has emerged as a promising strategy, particularly in the field of nanoparticle-mediated hyperthermia. This technique exploits the capacity of certain nanoparticles to convert external electromagnetic energy into heat, resulting in thermal stress in target tissues [13,14,15,16]. This heating method differs from traditional approaches because it generates homogeneous and controllable heat that spreads from the inside outward [17]. Over the last decade, several research groups have investigated different nanomaterials (gold nanorods, copper sulfide nanocrystals, tungsten oxide nanosheets, and different iron oxide nanoparticles) and electromagnetic energy sources (laser light and alternating magnetic fields) in an attempt to induce irreversible infertility in male animals [18,19,20,21,22,23,24].

Gold nanoparticles are known for their unique ability to convert light into heat, a property widely explored in cancer treatment through photohyperthermia (PHT) [25,26]. On the other hand, iron oxide nanoparticles are more biocompatible than gold nanoparticles. In this context, a maghemite nanoparticle covered with gold in a core–shell configuration could represent an interesting approach. Such nanoparticles have already demonstrated high efficiency in converting light into heat [27], and good in vitro [27,28] and in vivo [28] biocompatibility. Although several articles have investigated testicular PHT as a means of inducing infertility in male animals [18,19,20,21,23], all studies were conducted in mice, which have much smaller testes than the species of interest (mainly cats and dogs), so the methods developed may not be transferable. Since light penetration in biological tissues is limited [29], it is crucial to investigate testicular PHT in larger testicles, such as those of rats. Rat testicles are much larger than mouse testicles and are closer in size to those of cats. This way, using rats as an animal model to develop PHT-based methods for neutering stray animals may ensure a greater chance of transferability.

In the present study, we investigated the effects of testicular PHT using maghemite nanoparticles coated with gold and functionalized with citrate, combined with LED light irradiation, on reproductive parameters in Wistar rats as an animal model. We also described the biodistribution of these nanoparticles in vital organs (kidney, liver, spleen, and lungs) following intratesticular administration.

## 2. Results

Following administration of the magnetic fluid, the testicles became noticeably firmer and exhibited darker coloration. The dark color persisted until euthanasia, whereas the firmness gradually subsided and the organs returned to their original fibroelastic consistency.

**Figure 1:**
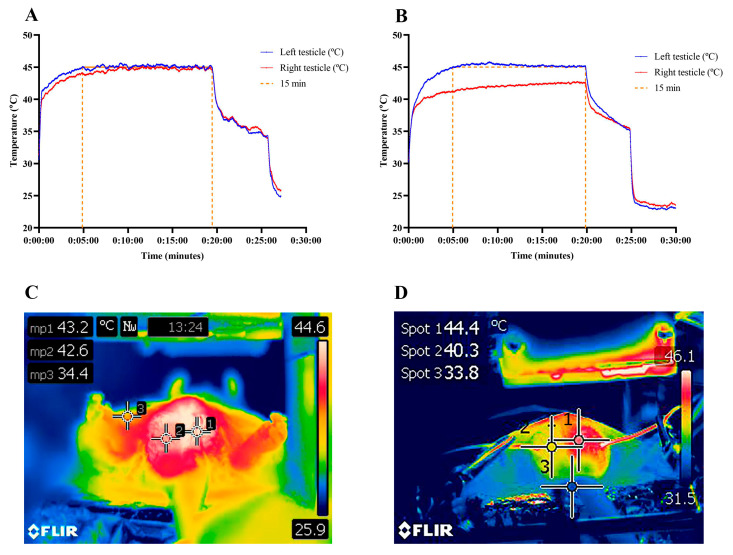
Heating curves (°C) and thermal imaging of testicular temperature in treated animals. (**A**,**C**) The animal received magnetic fluid injection and LED irradiation in both testicles (PHT group—1: left testicle; 2: right testicle; 3: right leg). (**B**,**D**) The animal received magnetic fluid injection only in the left testicle, with LED irradiation applied to both testicles (1: left testicle—PHT group; 2: right testicle—LED group; 3: tail).

The temperature recorded by the thermocouples before the LED equipment was turned on ranged from 29 to 31 °C. After turning on the device, the temperature increased progressively, reaching an average of 44.5 ± 1.4 °C in the PHT group and 43.5 ± 1.5 °C in the LED group. The average time to reach the target temperature was 4 min 38 s. Animals that received magnetic fluid injections in both testicles exhibited slight temperature variation between testicles (Figure 1A). In animals that received the magnetic fluid injection in only one testicle but were exposed to LED irradiation in both, the average temperature variation between the testicles was 1.8 °C (range 0.7–3.6 °C) (Figure 1B). Thermal imaging demonstrated that only the testicular region heated up during the PHT treatment (Figure 1C) and confirmed the temperature differential between testicles when only one testicle received the magnetic fluid injection (Figure 1D).

**Figure 2:**
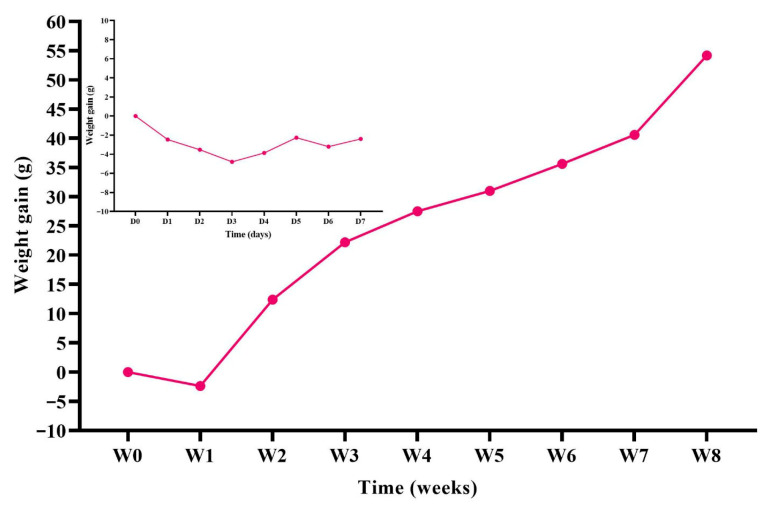
Mean weight gain of experimental animals following treatment. Inset: Weight variation during the first 7 days post-treatment. D: Day, W: week.

None of the animals exhibited signs of pain or discomfort following treatment, and no signs of inflammation (visible to the naked eye) were identified in the testicles or epididymides. The animals gained weight as expected (Figure 2), despite a short period (1 week) of stability immediately after treatment (Figure 2—inset).

**Figure 3:**
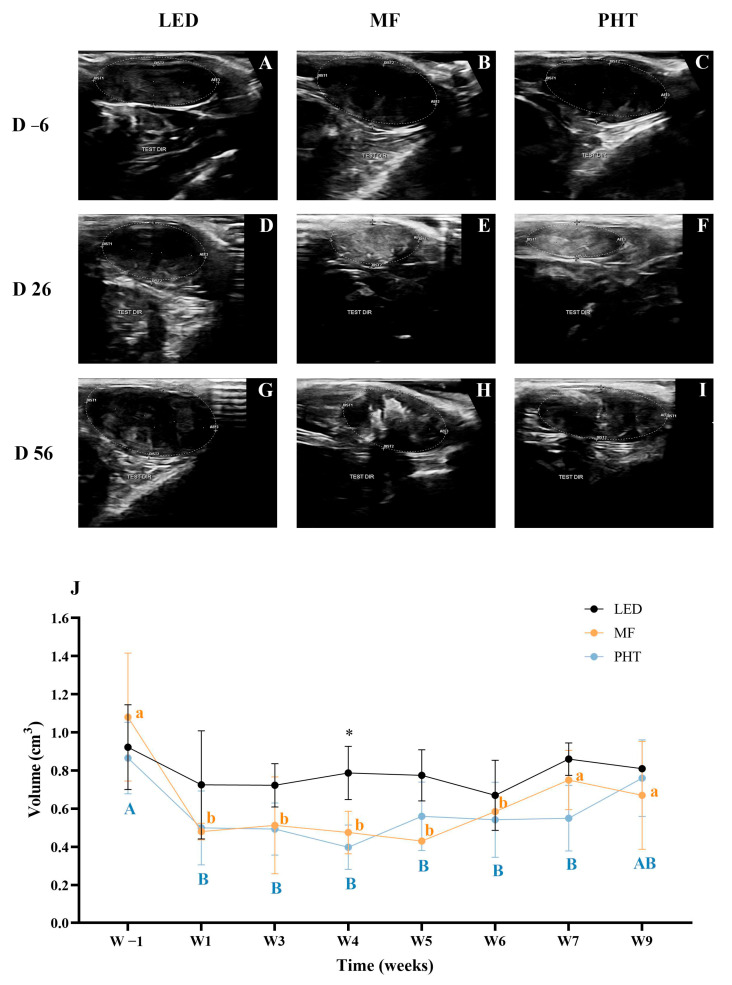
Representative testicular ultrasonographic images and longitudinal evaluation of testicular volume in the LED, MF, and PHT groups. (**A**–**I**) Testicular ultrasonographic images obtained 6 days before treatment (**A**–**C**), on day 26 post-treatment (**D**–**F**), and on day 56 post-treatment (**G**–**I**). (**J**) Mean testicular volume (cm^3^) measured by ultrasonography throughout the experimental period. A, B—Different uppercase letters indicate significant differences within the PHT group over time (*p* < 0.05). a, b—Different lowercase letters indicate significant differences between MF group over time (*p* < 0.05). Asterisk (*) indicates significant differences between the PHT and MF groups compared with the LED group at the same time point (*p* < 0.05).

Testicular ultrasonography conducted 6 days prior to the procedure revealed a homogeneous, hyperechoic echotexture in all animals (Figure 3A–C). By day 26, no alterations were observed in the LED group (Figure 3D). In contrast, the MF (magnetic fluid injection only) and PHT groups (Figure 3E,F) exhibited irregular margins, mixed echogenicity, and a heterogeneous, coarse echotexture. At 56 days post-treatment, testicular morphology remained unchanged in the LED group (Figure 3G), whereas irregular margins, mixed echogenicity, and heterogeneous parenchymal texture persisted in the MF and PHT groups (Figure 3H,I). Despite these morphological changes, no ultrasonographic signs of inflammation were observed and the alterations persisted throughout the post-treatment observation period.

The testicular volumes, measured from ultrasound images (Figure 3J), showed significant changes in the experimental groups. In the PHT group, testicular volume significantly reduced (*p* < 0.05) at weeks 1 (0.50 ± 0.19 cm^3^), 3 (0.49 ± 0.14 cm^3^), and 4 (0.40 ± 0.12 cm^3^) post-treatment, compared to the pre-treatment volume (0.87 ± 0.19 cm^3^). Similarly, in the MF group, testicular volume was significantly reduced (*p* < 0.05) at weeks 1 (0.48 ± 0.04 cm^3^), 3 (0.51 ± 0.25 cm^3^), 4 (0.48 ± 0.11 cm^3^), and 5 (0.43 ± 0.01 cm^3^) relative to the pre-treatment volume (1.08 ± 0.34 cm^3^). In contrast, no significant changes in testicular volume were observed in the LED group throughout the experimental period. At week 4, both the PHT and MF groups showed significantly lower testicular volumes (*p* < 0.05) compared to the LED group. However, by weeks 6, 7 and 8 post-treatment, testicular volumes were not significantly different (*p* > 0.05) from pre-treatment values, suggesting partial recovery.

Testicular weight was evaluated both in absolute values and relative to body weight (Table 1). In the LED control group, no significant changes were observed over time, with both absolute and relative weights remaining stable throughout the experimental period (*p* > 0.05). In the MF and PHT groups, relative testicular weight was significantly smaller on D28 and D56 compared with D7 (*p* < 0.05). Absolute testicular weight was also significantly lower (*p* < 0.05) on days 28 and 56 in the MF and PHT groups compared with the LED group and on D7 in the PHT group compared to the LED group. Additionally, the PHT group showed lower relative testicular weight than the LED group on D28 and D56 (*p* < 0.05). However, the only difference between the MF and PHT groups was a significantly smaller relative testicular weight in the PHT group on D7 (*p* < 0.05).

**Figure 4:**
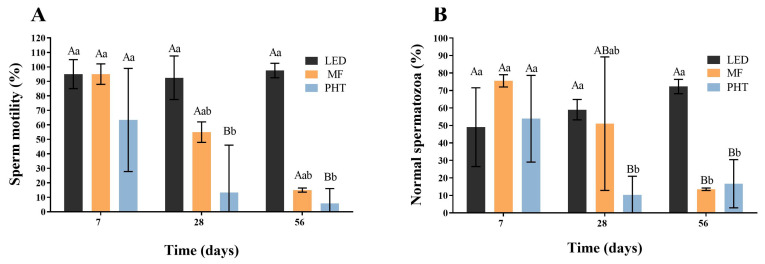
Sperm evaluation of epididymal tail washes in the LED, MF, and PHT groups on days 7, 28, and 56 post-treatment. (**A**) Sperm motility (%), (**B**) percentage of morphologically normal spermatozoa. A, B—Different letters indicate a significant difference within the same group over different time points (*p* < 0.05). a, b—Different letters indicate a significant difference between groups at the same evaluation time (*p* < 0.05).

The percentage of motile sperm (Figure 4A) obtained from the epididymis was significantly lower (*p* < 0.05) in the PHT group on days 28 and 56 post-treatment (13% and 6%, respectively) compared with 7 days post-treatment (63%) and with the LED group at the same time points (D28: 85%; D56: 95%). Similarly, a significant decrease (*p* < 0.05) in the percentage of morphologically normal spermatozoa (Figure 4B) was observed on days 28 and 56 post-treatment in the PHT group (10% and 17%, respectively) relative to 7 days post-treatment (54%) and to the LED group on the same evaluation days (D28: 62%; D56: 75%). The MF group showed lower percentages of motile sperm and sperm with normal morphology on days 28 and 56 post-treatment; however, these differences were not statistically significant (*p* > 0.05).

**Figure 5:**
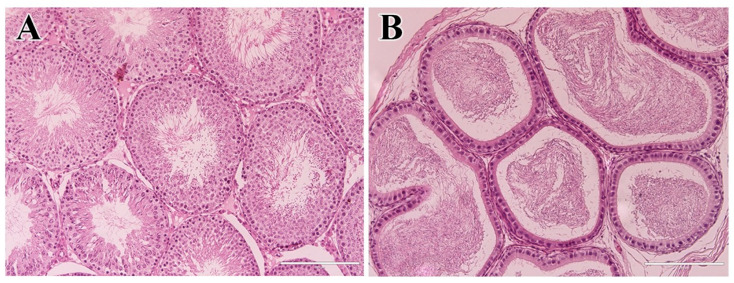
Histological sections of the LED group: (**A**) testicles; (**B**) epididymis. The structures of both organs were preserved at all evaluation time points, with spermatozoa present in the lumens of seminiferous and epididymal tubules. Scale bars = 200 µm.

Histological evaluation of testicles from the LED group revealed preserved seminiferous tubule architecture at all evaluation time points. The seminiferous tubules exhibited a well-organized epithelium presenting all germ cell types, including spermatogonia, spermatocytes, and spermatids, with spermatozoa present in the lumens (Figure 5A). The epididymal tubules were lined with pseudostratified columnar epithelium with stereocilia and contained lumens filled with spermatozoa (Figure 5B).

**Figure 6:**
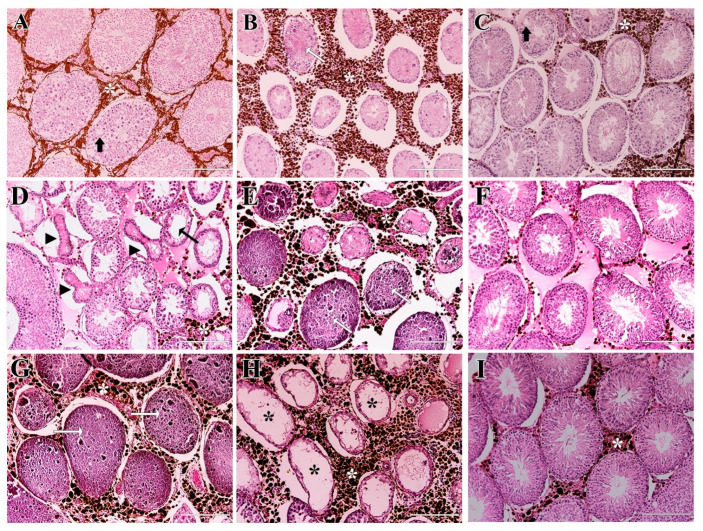
Histological sections of testicles from the PHT-treated group on days 7, 28 and 56 post-treatment. Day 7 (**A**–**C**): Seminiferous tubules exhibited initial vacuolization (thick black arrow), detachment of germinal cells, retraction, and coagulative necrosis (white arrow). Preserved tubules with spermatozoa in the lumen were also present (**C**). Day 28 (**D**–**F**): Seminiferous tubules were retracted and misshapen (black arrowhead) with very thin seminiferous epithelium (thin black arrow); coagulative necrosis (white arrow) was more pronounced. Intact seminiferous tubules were still visible in certain peripheral regions of the testicles (**F**). Day 56 (**G**–**I**): Seminiferous tubules presented extensive coagulative necrosis (white arrow) or complete loss of the seminiferous epithelium showing a wide empty lumen (black asterisk). Intact seminiferous tubules were still found in certain peripheral regions of the testicles (**I**). At all time-points, the presence of nanoparticle clusters was noticed in the interstitium (white asterisk). Scale bars = 200 µm.

Testicular histology of the PHT group revealed progressive degeneration from day 7 to day 56 post-treatment. On day 7 (Figure 6A–C), some seminiferous tubules exhibited initial vacuolization, detachment of germ line cells from the epithelium, tubular retraction, and coagulative necrosis. Tubular lumens were either empty or filled with cellular debris. By day 28 (Figure 6D–F), degeneration was more pronounced, with irregular shrunken tubules, a very thin seminiferous epithelium, and more evident coagulative necrosis. On day 56 (Figure 6G–I), degeneration was extensive, with most tubules presenting coagulative necrosis or complete destruction, showing only the basal lamina with a few lining cells and wide empty lumens. Although extensive degeneration was evident, tubules with a preserved seminiferous epithelium and spermatozoa in the lumen were still observed in certain peripheral regions of the testicles at all evaluation time points (Figure 6C,F,I). Agglomerates of nanoparticles were always visible in the interstitial space.

**Figure 7:**
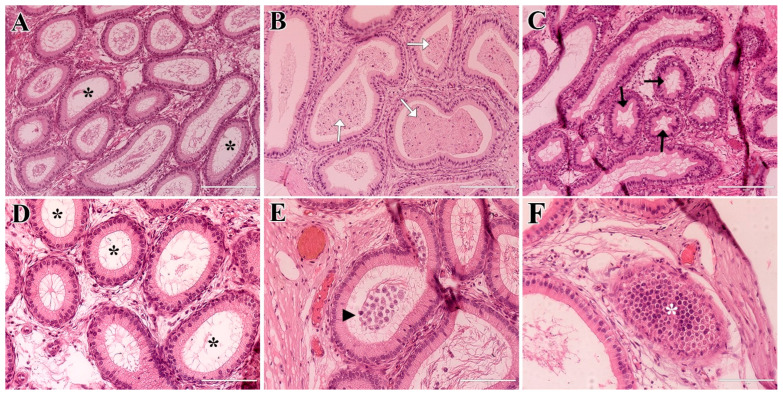
Histological sections of the epididymides from the PHT-treated group on days 7, 28, and 56 post-treatment. Day 7 (**A**–**C**): Some epididymal tubules contained spermatozoa in the lumen and some tubules were empty (black asterisk) or filled with cellular debris (white arrow). Empty tubules with cribriform alterations (black arrow) were also identified. Day 28 (**D**): Most tubules were empty (black asterisk). Day 56 (**E**,**F**): Some tubules contained lymphocytes in the lumen (black arrowhead) and lymphocytic infiltration was also observed in the interstitium (white asterisk). Scale bars: (**A**–**C**) = 200 µm, (**D**–**F**) = 100 µm.

In general, the epididymal histology of the PHT-treated group revealed epididymal tubules lined by pseudostratified columnar epithelial tissue with stereocilia. On day 7, most lumens were filled with spermatozoa, although some tubules were empty (Figure 7A). There were also some tubules with lumens filled with cellular debris (Figure 7B), and others showed cribriform alterations (Figure 7C). On day 28, most epididymal tubules were empty (Figure 7D) and lymphocytes were observed in the interstitium. On day 56, most tubules were empty or filled with cellular debris and some lumens contained lymphocytes (Figure 7E). Lymphocytic infiltration in the interstitium was also observed (Figure 7F).

**Figure 8:**
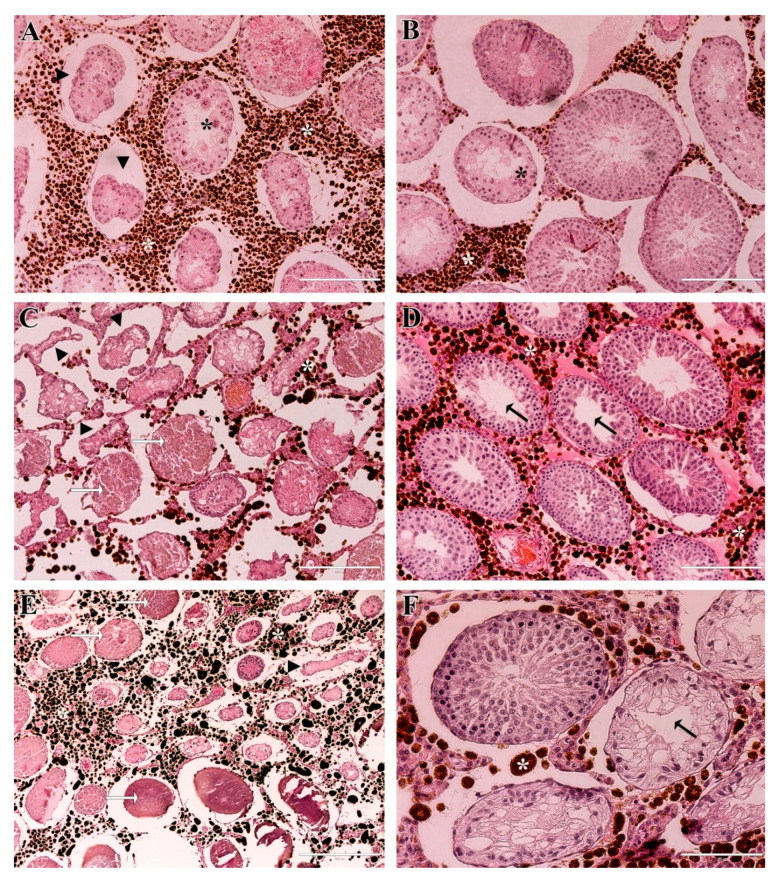
Histological sections of testicles from the MF-treated group on days 7, 28 and 56 post-treatment. Day 7 (**A**,**B**): Retracted seminiferous tubules (black arrowhead) containing multinucleated cells (black asterisk), along with preserved seminiferous tubules in areas with visible nanoparticle agglomerates (white asterisk). Day 28 (**C**,**D**): Retracted and misshapen tubules (black arrowhead) with very thin seminiferous epithelium and coagulative necrosis (white arrow), together with intact seminiferous tubules with empty lumens (black arrow). Day 56 (**E**,**F**): Predominance of retracted tubules and coagulative necrosis (white arrow), alongside intact and vacuolated seminiferous tubules with empty lumens (black arrow). Agglomerates of nanoparticles are seen in the interstitial tissue (white asterisk) Scale bars = (**A**–**E**) = 200 µm; (**F**) = 100 µm.

In the MF group, histological evaluation of the testes also revealed tissue damage. On day 7 post-treatment, some areas exhibited extensive damage, characterized by retracted seminiferous tubules lacking germ cells and the presence of multinucleated cells within the lumen (Figure 8A). Other regions, however, still showed preserved seminiferous tubules, with nanoparticles visible within the tissue (Figure 8B). By day 28, a larger number of seminiferous tubules displayed reduced diameter, tubular shrinkage, and areas of coagulative necrosis (Figure 8C), particularly in regions with higher nanoparticle accumulation, but preserved tubules were also present (Figure 8D). On day 56, histological lesions were similar to those observed on day 28 but more extensive, with some seminiferous tubules almost completely absent (Figure 8E). Nevertheless, intact seminiferous tubules were still observed in other areas of the testicular parenchyma (Figure 8F).

**Figure 9:**
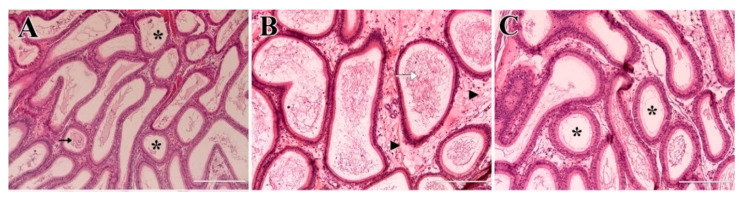
Histological sections of the epididymides from the MF group. (**A**): MF 7 days—Intact tubules, tubules without spermatozoa in the lumen (black asterisk), and tubules with the presence of cellular debris in the lumen (black arrow); (**B**): MF 28 days—presence of lymphocytic infiltrate in the interstitium (black arrowhead) and presence of spermatozoa in the lumen of the tubules (white arrow); (**C**): MF 56 days—tubules without spermatozoa in the lumen (black asterisk). Scale bars: (**A**) = 400 µm; (**B**,**C**) = 200 µm.

Epididymal histology of the MF group revealed that, on day 7 post-treatment, epididymal tubule lumens were empty or filled with exfoliated material (Figure 9A). On days 28 (Figure 9B) and 56 (Figure 9C), tubules presented spermatozoa in the lumen, although some empty tubules were also found.

The extent of testicular damage and the corresponding Johnsen scores for the PHT and MF groups are presented in Table 2. In the PHT group, the damaged area varied from 23 to 94% across all evaluation time points, whereas in the MF group it ranged from 29 to 93%. A significant increase (*p* < 0.05) in damaged area was observed only in the MF group between day 28 and day 56 post-treatment. No differences were observed between the MF and PHT groups at any time point. Johnsen score was significantly higher (*p* < 0.05) in the MF group on day 28 than on days 7 and 56 and was also higher than the PHT group on day 28. In PHT group, Johnsen score was significantly higher (*p* < 0.05) on day 56 compared to days 7 and 28.

The liver, kidneys, spleen, and lungs exhibited normal macroscopic appearance in all groups. The only statistical difference observed in the organ relative weight (Table 3) of animals that received bilateral intratesticular injections of nanoparticles was a significant decrease (*p* < 0.05) in liver relative weight on days 28 and 56 compared to day 7.

**Figure 10:**
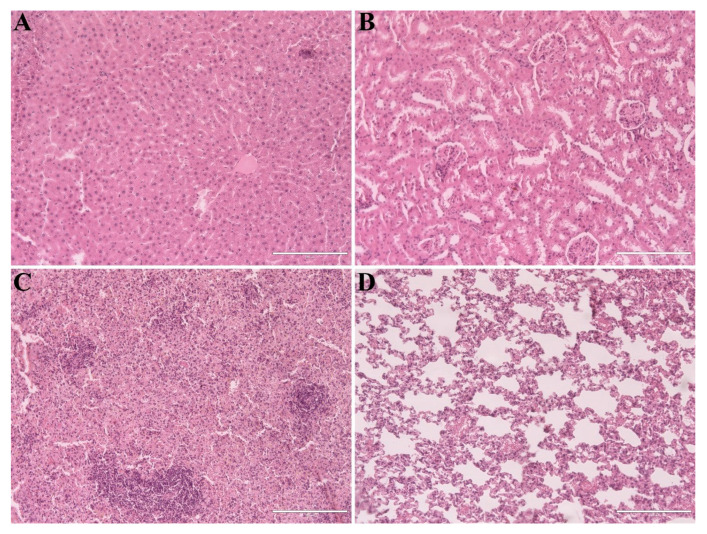
Histological sections showing the general appearance of organs from animals treated with bilateral testicular photohyperthermia mediated by γ-Fe_2_O_3_@Au nanoparticles on day 56 post-treatment. All organs presented normal morphology. (**A**) Liver; (**B**) kidneys; (**C**) spleen; (**D**) lung. Scale bar = 200 μm.

Histopathological analysis of the liver, kidneys, spleen, and lungs revealed no morphological alterations in either the PHT or MF groups euthanized on days 7, 28, and 56 (Figure 10). The liver (Figure 10A) exhibited preserved hepatic parenchyma with normal lobular architecture; both the central veins and hepatocyte cords maintained normal histological features, with no relevant alterations across treatment groups. The renal cortex (Figure 10B) displayed typical morphology, with preserved renal corpuscles and convoluted tubules. The spleen (Figure 10C) also showed typical morphology, with preserved structure of the white and red pulp, trabeculae, capsule, reticular cells, and splenic macrophages at all time-points. The lungs (Figure 10D) displayed thickening of the alveolar septa, particularly in areas adjacent to the bronchioles, irrespective of treatment or evaluation day. Despite this observation, the alveolar lumens remained preserved, with no evidence of pulmonary congestion, inflammatory infiltrates or respiratory distress.

**Figure 11:**
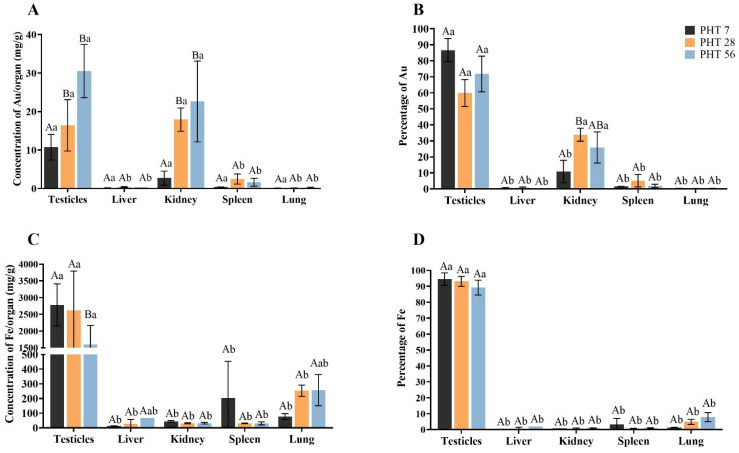
Distribution of gold (Au) and iron (Fe) elements present in the γ-Fe_2_O_3_@Au nanoparticles in the organs (testicles, liver, kidneys, spleen, and lungs) of animals from the PHT group that received bilateral nanoparticle injections. (**A**): Au concentration (mg/g); (**B**): percentage of total Au in the evaluated organs; (**C**): Fe concentration (mg/g); (**D**) percentage of total Fe in the evaluated organs. A, B—Different letters indicate a significant difference (*p* < 0.05) between time points within the same organ. a, b—Different letters indicate a significant difference (*p* < 0.05) between organs at the same evaluation time.

Biodistribution of gold (Au) and iron (Fe) derived from γ-Fe_2_O_3_@Au nanoparticles was only assessed in animals from the PHT group that received bilateral intratesticular injections. Au concentrations were consistently higher in the testicles and kidneys than in the liver, spleen, and lungs (*p* < 0.05; Figure 11A). Over time, Au accumulation in the testicles and kidneys became more pronounced, with significantly higher levels detected on days 28 and 56 compared to day 7 (*p* < 0.05). When expressed as percentages, the testicles accounted for the highest proportion of Au on day 7, differing significantly from all other organs (*p* < 0.05). On days 28 and 56, both the testicles and kidneys contributed the largest fractions of Au, with significantly higher percentages than the other organs (*p* < 0.05; Figure 11B). Fe levels were also predominantly higher in the testicles compared with other organs at all time points. Although absolute Fe levels in the testicles decreased significantly by day 56 (*p* < 0.05; Figure 11C), the relative proportion of Fe remained consistently above 90% throughout the evaluation period (Figure 11D).

## 3. Discussion

Over the past 50 years, numerous attempts have been made to develop non-surgical castration methods, but none have achieved complete success. In recent years, nanotechnology has contributed to the development of novel strategies. While some studies have investigated the toxic effects of certain nanomaterials [30,31], others have invested in nanoparticle-mediated hyperthermia using various nanocomposites [18,19,20,21,22,23,24]. In the present study, we investigated PHT mediated by maghemite–gold core–shell nanoparticles (γ-Fe_2_O_3_@Au) surface-functionalized with citrate activated by NIR LED light (808 nm). Testicular heating occurred as expected, reaching approximately 45 °C within 5 min and remaining stable for 15 min. This thermal exposure induced progressive testicular damage, initially characterized by vacuolization and detachment of the seminiferous epithelium, followed by coagulative necrosis and extensive degeneration, accompanied by the absence of spermatozoa in the epididymal tubules. Sperm recovered from the cauda epididymis, when present, exhibited markedly reduced motility (<13%) and normal morphology (<17%), reinforcing the impact of the treatment on reproductive parameters. These findings are consistent with previous reports in murine models using different nanomaterials and photothermal therapy. For example, similar testicular temperatures (~45 °C) and histopathological findings were observed when mice were treated with PHT using intratesticular injections of gold nanorods combined with 808 nm laser irradiation for 10 min [18], as well as with plasmonic copper sulfide nanocrystals or tungsten oxide nanosheets combined with 980 nm laser irradiation for 5 min [19,20]. Comparable results were also obtained using cysteine-coated copper sulfide nanosheets with 1064 nm laser irradiation for 40 s; however, in this case testicular temperature reached 54 °C [23].

Nevertheless, in the present study some intact seminiferous tubules were observed in the peripheral regions of PHT-treated testicles, irrespective of the post-procedure time point (7, 28 or 56 days). This finding suggests that heating in these areas was insufficient, which may have been due to either light irradiation not reaching the opposite side of the testis or nanoparticles not being homogeneously spread in the tissue. Unaffected regions were not described in previous studies investigating testicular PHT in mice [18,19,20]. However, it is important to remember that mouse testicles are much smaller than those of rats, not to mention the species of interest (cats and dogs). Light penetration in biological tissues is limited [32], and both the depth of penetration and absorption vary with wavelength and power density. For example, penetration has been described as 3.4 cm for 808 nm at 1 mW/cm^2^ and 2.2 cm for 980 nm at 1 mW/cm^2^ [29]. Other studies report a much lower penetration, such as 2–3 mm in the 650–950 nm wavelength range [33]. Overcoming this limitation is essential if the PHT method is to be considered a feasible method for neutering male stray animals.

Attempting to overcome the obstacle of light penetration, Wang et al. [23] developed a PHT method using a nanocomposite excitable by light in the second near infrared window (NIR-II; 1000–1700 nm), which penetrates more deeply into biological tissues. The authors reported that after intratesticular injection of cysteine-coated copper sulfide nanosheets and laser irradiation (1064 nm) for 40 s, mice testicles reached 54 °C, resulting in decreased plasma testosterone levels and no pups born in a fertility test 60 days post-treatment. However, such high temperatures (>50 °C), even for short durations, may result in undesirable side effects, including pain, organ inflammation, abscess formation, or infection [34], which were not evaluated in the study. Using a different strategy, Yu et al. [35] developed a system containing human heavy chain ferritin (HFn) nanocarriers loaded with aggregation-induced emission luminogens. Upon 808 nm laser irradiation, the nanocarriers emitted NIR-II light inside the target tissue. The authors tested the photothermal effect of their system in male mice intravenously injected with the nanocarriers and submitted to laser irradiation (1.0 W/cm^2^) 24 h after injection. Testicular temperature reached 45 °C within approximately 1 min and was maintained for 10 min. Histological evaluation revealed testicular damage and absence of sperm on the epididymis up to 60 days post-treatment. Although promising, this approach is limited by the requirement of a 24 h interval between injection and light irradiation, constituting a hindrance.

Another potential strategy is to induce nanoparticle-mediated hyperthermia using an alternating magnetic field, a technique known as magnetohyperthermia (MHT). This approach has the advantage that magnetic fields have no penetration limit in biological tissues [36]. Previous studies have demonstrated successful infertility induction in mice [21], rats [22,24] and cats [37] treated with testicular MHT. However, MHT requires more complex equipment and infrastructure than PHT, which may limit practical application.

An unexpected and noteworthy finding of the present study was that intratesticular injection of nanoparticles alone (MF group) also induced testicular damage. These γ-Fe_2_O_3_@Au nanoparticles had no toxic effects both in vitro (in keratinocytes and fibroblasts) [27] and in vivo in mice and dogs [28]. However, in the present study, the histological alterations in testicular tissue observed in the MF group were similar to those detected in the PHT group up to 56 days. It would be very practical if an intratesticular injection of nanoparticles, by itself, could impair spermatogenesis to the point of causing infertility. Indeed, previous studies investigating intratesticular injections of different nanoparticles (gold nanoparticles [31]; silver nanoparticles [30]) showed some toxic effects on spermatogenesis; however, these were always moderate and reversible. In the present study, a complementary experiment revealed that the testicular damaged area was reduced from 85.5 ± 5.2% to 47.3 ± 13.9% on day 70 and then to 3.5 ± 4.4% on day 90 after intratesticular injection of γ-Fe_2_O_3_@Au nanoparticles. The Johnsen score also improved to 5.9 ± 0.4 and 8.5 ± 0.2 respectively on days 70 and 90, together with 100% sperm motility and over 65% morphologically normal spermatozoa. In contrast, PHT-treated animals still showed 39.9 ± 36.9% and 36.8 ± 29.8% testicular tissue damage, respectively, on days 70 and 90 post-procedure, and respective Johnsen scores of 5.0 ± 1.2 and 5.9 ± 0.7, together with 35–50% sperm motility and normal morphology. Although the recovery of reproductive parameters was less pronounced in the PHT group, these findings highlight that, for a PHT-based method to be developed for male animal neutering, further refinement and optimization of the technique will be necessary.

Besides the reproductive effects of testicular PHT treatment, the present work describes the biodistribution of the elements of the nanoparticles (Au and Fe) in vital organs. Au was predominantly found in the testicles at all evaluation time points (>60%), but was also detected in the kidneys, especially on days 28 and 56 (34 and 26%, respectively). Previous studies have demonstrated that gold nanoparticles (10–250 nm) are typically eliminated via renal excretion [38,39,40]. The increasing proportion of gold in the kidneys over time suggests renal clearance of this element. Using two intratesticular injections of gold nanoparticles functionalized with PEG, Coimbra et al. [31] demonstrated signals of oxidative stress in the animal kidneys and liver, albeit without clinical symptoms of intoxication. The highest Fe content was also found in the testicles at all time points. Although the absolute values decreased, particularly by day 56, the relative proportion remained consistently high (>85%), indicating that the testicles were the main site of Fe retention. Previous studies using citrate-functionalized iron nanoparticles reported high Fe accumulation in the liver and spleen up to 60 days after intratesticular [22] or intravenous administration [21]. It is important to note that the composition, synthesis method, particle size and functionalization of each nanomaterial interfere with their accumulation sites and clearance routes [40].

Importantly, the levels of gold and iron detected in other organs, including the liver, spleen, and lungs, were negligible at all evaluated time points. Consistent with these findings, macroscopic inspection and histopathological analyses revealed preserved structural integrity of the liver, kidneys, spleen, and lungs, with no clinical signs of systemic toxicity observed. The alveolar septal thickening observed in the lungs of all animals, irrespective of treatment, was probably due to the inhalation of dust from the wood shavings used as bedding [41]. Together, these results support the relative safety of the intratesticular administration route and reinforce the potential of nanoparticle-mediated hyperthermia approaches for reproductive control. Nevertheless, further studies are warranted to optimize and refine this strategy.

## 4. Material and Methods

### 4.1. Nanoparticles

Gold-coated maghemite nanoparticles (γ-Fe_2_O_3_@Au) surface-functionalized with citrate were produced according to the improved chemical method described by Coelho et al. [27]. In this procedure, citrate-capped maghemite nanoparticles were first prepared by coprecipitation of iron salts followed by oxidation. Briefly, an aqueous solution (50 mL) containing Fe^3+^ (50 mmol), Fe^2+^ (25 mmol), and HCl (20 mmol) was rapidly added to an NH_4_OH aqueous solution (250 mL, 1 mol/L) under vigorous stirring (1000 rpm) at room temperature for 30 min, producing magnetite (Fe_3_O_4_) nanoparticles. The precipitate was magnetically separated and washed with water until reaching neutral pH. The material was then acidified with HNO_3_ (0.5 mol/L) and oxidized to maghemite (γ-Fe_2_O_3_) by boiling the suspension in Fe(NO_3_)_3_ solution (0.5 mol/L) for 30 min. Surface functionalization was achieved by treating the nanoparticles with trisodium citrate solution (1.0 mol/L) at 80 °C for 30 min using a citrate-to-iron molar ratio of 0.1. The resulting particles were magnetically collected, washed with acetone, redispersed in water, and the pH was adjusted to 7.0. Gold coating was performed by the reduction of Au^3+^ ions onto the citrate-capped maghemite surface using NaBH4 as a reducing agent. The maghemite dispersion (80 µL, 400 mg/mL) was diluted in water (80 mL) and mixed under sonication with HAuCl4 solution (360 µL, 1 wt%). After 10 min, NaBH_4_ solution (300 µL, 0.3 mol/L in ethanol) was added and the mixture was sonicated for an additional 10 min to promote gold deposition. The HAuCl_4_/NaBH_4_ reduction cycle was repeated four times.

The morphology and size of the synthesized nanoparticles were investigated by high-resolution transmission electron microscopy (HRTEM) using a JEOL 1100 microscope (Jeol, Tokyo, Japan). Magnetic measurements at room temperature were performed using a vibrating sample magnetometer (VSM, ADE Technologies, model EV7, ADE Technologies Inc., Westwood, MA, USA). Magnetization curves were recorded by applying magnetic fields up to 1.8 T. The elemental composition of the samples was determined by inductively coupled plasma optical emission spectrometry (ICP-OES) using a PerkinElmer Optima 8000 instrument (PerkinElmer, Waltham, MA, USA). Prior to analysis, the nanoparticle samples were digested in a HNO_3_:HCl solution (1:1 *v*/*v*) to ensure complete dissolution. Hydrodynamic diameter (DH), polydispersity index (PDI), and zeta potential were measured by dynamic light scattering and electrophoretic mobility using a Zetasizer Nano ZS instrument equipped with an MPT-2 autotitrator (Malvern Instruments, Worcestershire, UK). Measurements were performed using aqueous dispersions of the nanoparticles and NaOH and HNO_3_ as titrant solutions.

**Figure 12:**
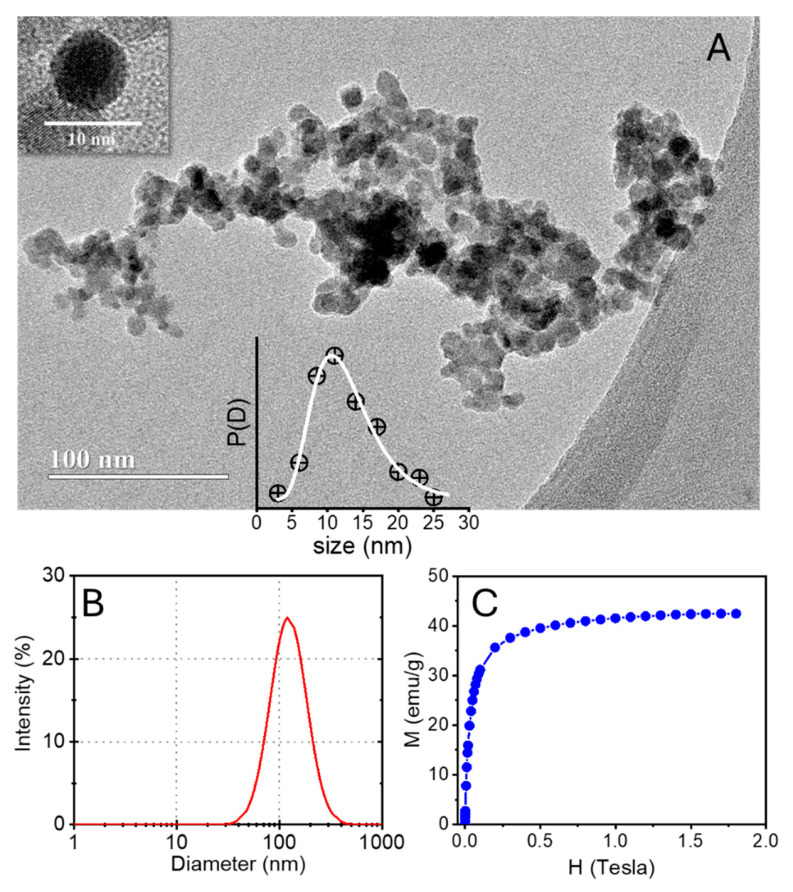
(**A**) Representative HRTEM micrograph of γ-Fe_2_O_3_@Au nanoparticles. The bottom inset shows the particle size distribution obtained from HRTEM images and fitted with a log-normal distribution. The upper inset presents a magnified HRTEM image of a single nanoparticle, confirming the typical γ-Fe_2_O_3_@Au core–shell structure. (**B**) Dynamic light scattering (DLS) size distribution of the gold-coated maghemite nanoparticles in aqueous dispersion at pH 7. (**C**) Magnetization (M) as a function of the applied magnetic field (H) for gold-coated maghemite nanoparticles measured at room temperature.

The representative TEM micrograph indicates that the gold-coated nanoparticles were mostly spherical in shape (Figure 12A). Analysis of the size distribution derived from the TEM images provided an average particle diameter of 12.1 nm and a polydispersity parameter of 0.30, as determined from a log-normal fitting of the corresponding histogram (inset at the bottom of Figure 12A). The magnified HRTEM image of a gold-coated nanoparticle (inset at the top of Figure 12A) confirms the formation of a typical γ-Fe_2_O_3_@Au core–shell nanostructure. Dynamic light scattering (DLS) measurements revealed a hydrodynamic diameter of ~157 nm with a polydispersity index (PDI) of 0.18 (Figure 12B) at pH 7, indicating a relatively narrow size distribution of the nanoparticles in aqueous suspension. At this pH, the measured zeta potential was approximately −40 mV, which was attributed to the ionization of citrate groups adsorbed on the nanoparticle surface. Such highly negative zeta potential values suggest strong electrostatic repulsion between particles, contributing to the good colloidal stability of the dispersion. Figure 12C shows the magnetization (M) as a function of the applied magnetic field (H) for gold-coated maghemite nanoparticles in the positive quadrant of the magnetization loop (increasing and decreasing field). The magnetization increased rapidly at low fields and gradually approached saturation at higher fields, which is typical for ferrimagnetic nanoparticles. The saturation magnetization reached approximately 42–43 emu/g, slightly lower than that of bulk γ-Fe_2_O_3_@Au, likely due to surface spin disorder and the presence of the diamagnetic gold coating, which increased the total mass without contributing to the magnetic moment. Elemental analysis by ICP-OES indicated that the gold content in the γ-Fe_2_O_3_@Au nanoparticles was 10.1% (*w*/*w*). In addition, no hysteresis was verified, suggesting that the nanoparticles were close to superparamagnetic behavior at room temperature.

### 4.2. Light Source and Luminous Power Control

An LED (808 nm) was used as the light source and was connected to a temperature control system.

Initially, an in vitro heating test of the nanoparticles (γ-Fe_2_O_3_@Au) was performed using LED irradiation without a controller. Approximately 1 mL of the sample was added to a 10 mL beaker and exposed to LED light at maximum power. The sample temperature reached 67.5 °C in 3 s without observable evaporation. Subsequently, a test using the control system was performed to calibrate the equipment and maintain the temperature at 45 °C.

Heating of the testicles was achieved through the absorption of light energy by the nanoparticles, which converted it into thermal energy, thereby increasing the temperature of the compounds and testicles. The surface temperature of the testicles was measured using type K thermocouple sensors (chromel/alumel junction). The measured temperature was sent to a specific microcontroller circuit to regulate the current of the LED, adjusting the emitted light power to maintain the testicular temperature at approximately 45 °C.

### 4.3. Animals and Experimental Design

A total of 21 male Wistar rats (10 weeks old) were used. The animals were housed in groups of 4 to 5 per cage under controlled conditions: 25 °C, 12 h light/12 h dark cycle, and with ad libitum access to tap water and commercial rat food (Nutrina^®^, São Sebastião, DF, Brazil). All animal experimental procedures were approved by the Ethics Committee on Animal Use of the University of Brasilia (protocol number, UnBDOC 71/2019).

The experiment was conducted based on the number of testicles (*n* = 36 testicles; 18 animals) and allocated into groups according to the treatment received as follows: (1) the PHT group (*n* = 24 testicles), which received an injection of the magnetic fluid (γ-Fe_2_O_3_@Au) followed by exposure to LED light to induce testicular PHT; (2) the MF group (*n* = 6 testicles), which received only an injection of magnetic fluid (γ-Fe_2_O_3_@Au); and (3) the LED group (*n* = 6 testicles), which was exposed only to LED light irradiation without magnetic fluid injection. To ensure that the testicles in the LED group were exposed to light emission comparable to that of the PHT group, 6 animals received an intratesticular injection of magnetic fluid in one testicle, and both testicles were subsequently exposed to the LED irradiation at the same light intensity. In addition, the testicles in each group were subdivided into 3 subgroups, according to the evaluation time (7, 28, and 56 days post-treatment) as follows: PHT-D7 (*n* = 8), PHT-D28 (N = 8), and PHT-D56 (*n* = 8); MF-D7 (*n* = 2), MF-D28 (*n* = 2), and MF-D56 (*n* = 2); and LED-D7 (*n* = 4), LED-D28 (*n* = 4), and LED-D56 (*n* = 4).

**Figure 13:**
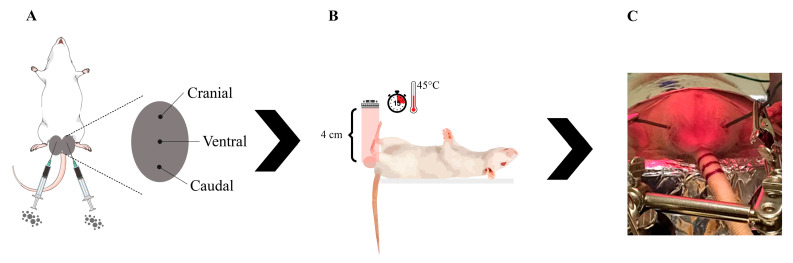
Schematic representation of the experimental setup during treatment. (**A**): Intratesticular injection was performed at 3 sites (cranial, middle, and caudal), with 50 μL of γ-Fe_2_O_3_@Au magnetic fluid administered at each site. (**B**): Animal positioning under LED irradiation. Once the testicular temperature reached 45 °C, it was maintained for 15 min. (**C**): Picture of animal receiving LED irradiation, showing the thermocouples positioned on the lateral surface of each testicle for temperature monitoring.

On the day of treatment (D0), each animal was weighed, administered morphine (morphine sulfate, 2 mg/kg IM) and anesthetized with an intraperitoneal injection of ketamine (Cetamin^®^, Syntec, 60 mg/kg, Barueri, Brazil) and xylazine (Calmiun^®^, Agener União, 8 mg/kg, São Paulo, Brazil). After shaving and cleaning the scrotum, testicles assigned to the PHT and MF groups were injected with 150 μL of γ-Fe_2_O_3_@Au magnetic fluid at 3 distinct points (cranial, middle, and caudal) (Figure 13A). The volume used was determined by ex vivo rat testicle assays and has been used in previous studies [22,24]. Testicles assigned to the LED group did not receive any injection. Subsequently, testicles assigned to the PHT and LED groups were positioned in the equipment so that the ventral surface was exposed to LED irradiation at a distance of 4 cm (Figure 13B). A thermocouple sensor was positioned on the lateral surface of each testicle (Figure 13C) to monitor temperature and transmit data to the microcontroller to maintain the temperature of the treated testicles at 45 °C for 15 min. An FLIR^®^ T420 thermographic camera (FLIR Systems, Wilsonville, OR, USA) was used to monitor temperature changes in the testicular region during the procedure. After treatment, all animals received a dose of analgesic and anti-inflammatory (meloxicam, 1 mg/kg SC).

### 4.4. Experimental Endpoints

The animals were visually evaluated for signs of pain, according to the facial and body expression scale described by Sotocinal et al. [42], as well as for signs of inflammation every 2 h during the first 12 h and daily throughout the first week. Behavior and general appearance were monitored weekly throughout the experiment. Animal body weight was recorded daily during the first 7 days and weekly thereafter until the end of the experiment.

All animals underwent testicular ultrasonographic evaluation before treatment and weekly after treatment to assess the presence, size, and general appearance of the testicles and epididymides. A veterinary color Doppler ultrasound device (model Z5 vet, Mindray, Shenzhen, China) equipped with a linear multifrequency (7.5 to 10 MHz) probe was used.

Animals were euthanized at the predetermined evaluation time according to their respective groups by anesthetic overdose. After euthanasia, both testicles and epididymides, as well as the liver, spleen, kidneys, and lungs, were removed. The testicles were separated from the epididymides, measured, weighed, and fixed in Bouin’s solution for 24 h for histopathological analysis. The length and width measurements (cm) of the testicles were used to determine their volume. For this, the mean of 2 mathematical equations was used: the cylinder VOLC = [(WIDTH/2)^2^ × Π × (LENGTH)] and the prolate spheroid VOLP = [4/3 × Π × (WIDTH/2)^2^ × (LENGTH/2)], as previously described [24]. Relative testicular weight was determined by dividing the weight of each testicle by the animal’s body weight, and the result was expressed as a percentage.

The cauda epididymides were macerated in 2 mL of prewarmed saline solution (37 °C). A drop of the resulting suspension was immediately placed on a slide to determine the percentage of motile spermatozoa under a light microscope (Model E100, Nikon Instruments, Shanghai, China). Subsequently, 1 mL of the remaining suspension was mixed with 1 mL of 10% formalin solution to evaluate sperm morphology using phase-contrast microscopy (Nikon Eclipse Ci, Tokyo, Japan). The caput and corpus of the epididymides were fixed in Bouin’s solution for 24 h and processed for histopathological analysis.

The remaining organs were weighed and divided into 2 portions; one portion was fixed in 10% formalin for 24 h for histopathological analysis and the other was frozen for subsequent nanoparticle quantification. Relative organ weight was calculated by dividing the weight of each organ by the animal’s body weight and the result was expressed as a percentage.

### 4.5. Histological Processing and Analysis

The organs designated for histopathological analysis were dehydrated in ethanol clarified with xylene and embedded in paraffin. Sample sections (5 µm thick) were stained with hematoxylin and eosin (H&E), examined under a light microscope (Nikon Eclipse Ci-S, Tokyo, Japan) and photographed using an EVOS™ FL Auto Imaging System (Thermo Fisher Scientific, Waltham, MA, USA).

The histopathological parameters evaluated in the testicles and epididymis included the general structure of the seminiferous and epididymal tubules, the interstitial tissue, and the presence of spermatozoa in the lumen. The other organs (spleen, liver, kidneys, and lungs) were evaluated for their general structure and overall integrity.

To further evaluate testicular damage, the entire cross-sectional area of each testis was scanned using an EVOS™ FL Auto Imaging System. The acquired images were analyzed with software ImageJ 1.54g [43] to determine the total sectional area and calculate the percentage of damaged tissue. Spermatogenesis was assessed according to the criteria described by Johnsen [44], which provide a quantitative index of tubular damage. The Johnsen scoring system is a 10-point scale reflecting the most advanced germ cell type present within the seminiferous tubules. A score of 10 indicates complete and normal spermatogenesis, whereas a score of 1 indicates the complete absence of germ cells. Johnsen scoring was performed on all seminiferous tubules identified in 3 different histological sections per testis.

### 4.6. Biodistribution of Nanoparticles in Organs

For the biodistribution of γ-Fe_2_O_3_@Au, the organ samples previously frozen after euthanasia were placed in 15 mL Falcon tubes and digested in 2 mL of 70% nitric acid for 48 h. After digestion, the tubes were subjected to a short-duration ultrasonic water bath, followed by a 30 min resting period. Subsequently, 20 µL of the sample was diluted in 10 mL of milli-Q water. Finally, the quantification of gold (Au) and iron (Fe) was performed by Inductively Coupled Plasma Mass Spectrometry (iCAP™ Q ICP-MS Thermo Scientific™, Waltham, MA, USA). The values detected by the equipment were used to calculate the concentration of Au and Fe per gram of organ tissue according to the calculations detailed in document ISM02.3, Exhibit D of the United States Environmental Protection Agency [45], as follows:

$$Concentration\;\left(\frac{mg}{kg}\right)=C\times \frac{Vf}{W\times S}\times \frac{DF}{1000}$$

*C* = value detected in μg/L (the average across all replications);

*Vf* = final digested volume (mL);

*W* = initial aliquot (g);

*S* = % solid/1000;

*DF* = dilution factor;

% Solid = weight of dried solid (g)/total weight of solid (g) × 100.

### 4.7. Statistical Analysis

All data were tested for normality using the Shapiro–Wilk test. The data were compared between the experimental groups by analysis of variance (ANOVA) and the Tukey test using the GraphPad prism 8.0.2 statistical program (GraphPad Software: San Diego, CA, USA), with a significance level of 5% (*p* < 0.05).

## 5. Conclusions

In conclusion, the approach investigated in this study, combining citrate-functionalized, gold-coated maghemite nanoparticles (γ-Fe_2_O_3_@Au) with 808 nm LED irradiation to induce testicular PHT, effectively disrupted spermatogenesis and induced testicular tissue damage. Notably, no clinically relevant side effects or signs of systemic toxicity were observed, supporting the relative safety of the intratesticular administration route. However, the presence of preserved seminiferous tubules in the peripheral regions of the testes, likely due to incomplete light penetration or nonhomogeneous nanoparticle distribution in thicker tissues such as rat testes, highlights the need for further optimization of this strategy or investigation of alternative approaches. Together, these findings demonstrate the potential of nanoparticle-mediated PHT to induce infertility in male animals, while emphasizing the importance of technique refinement to achieve uniform and permanent testicular damage.

## Figures and Tables

**Table 1:** Mean (±SD) absolute and relative testicular weights of animals from the LED, MF and PHT groups on days 7, 28 and 56 post-treatment.

Absolute Weight (g)	Day 7	Day 28	Day 56
LED	1.40 ^Aa^ ± 0.36	1.63 ^Aa^ ± 0.06	1.55 ^Aa^ ± 0.10
MF	1.15 ^Aab^ ± 0.03	1.07 ^Ab^ ± 0.32	0.98 ^Ab^ ± 0.08
PHT	1.04 ^Ab^ ± 0.16	0.83 ^Ab^ ± 0.10	1.01 ^Ab^ ± 0.26
**Relative Weight (%)**			
LED	0.48 ^Aab^ ± 0.14	0.50 ^Aa^ ± 0.04	0.45 ^Aa^ ± 0.02
MF	0.54 ^Aa^ ± 0.01	0.31 ^Bab^ ± 0.09	0.27 ^Bb^ ± 0.03
PHT	0.40 ^Ab^ ± 0.05	0.27 ^Bb^ ± 0.02	0.31 ^Bb^ ± 0.10

Absolute weight = measured testicular mass in grams. Relative weight = testicular weight/body weight × 100. A, B—Different letters indicate a significant difference within a row (*p* < 0.05). a, b—Different letters indicate a significant difference within a column (*p* ≤ 0.05).

**Table 2:** Testicular damaged area (%) and Johnsen score for MF and PHT groups on days 7, 28, and 56 post-treatment.

	% Damage (Mean ± SD)		Johnsen Score (Mean ± SEM)	
Day	MF Group	PHT Group	MF Group	PHT Group
7	65.5 ± 7.1 ^ABa^	55.5 ± 12.8 ^Aa^	3.5 ± 0.6 ^Aa^	2.7 ± 0.5 ^Aa^
	(61–76)	(43–78)	(2.9–4.0)	(1.1–4.3)
28	59.0 ± 21.5 ^Aa^	66.9 ± 24.2 ^Aa^	5.3 ± 2.7 ^Ba^	2.6 ± 0.5 ^Ab^
	(29–75)	(23–94)	(2.6–7.9)	(1.2–4.2)
56	85.5 ± 5.2 ^Ba^	65.0 ± 14.0 ^Aa^	3.1 ± 0.7 ^Aa^	3.6 ± 0.9 ^Bb^
	(81–93)	(50–81)	(2.4–3.7)	(1.1–6.4)

A, B—Different letters indicate a significant difference (*p* < 0.05) among different days for the same treatment group. a, b—Different letters indicate a significant difference (*p* < 0.05) among groups on the same evaluation day.

**Table 3:** Mean (±SD) relative weight (%) of the liver, kidneys, spleen, and lungs in animals that received bilateral intratesticular injections of nanoparticles on days 7, 28 and 56 post-treatment.

	Relative Weight (%)		
Organ	Day 7	Day 28	Day 56
liver	3.42 ± 0.23 ^A^	2.99 ± 0.08 ^B^	2.94 ± 0.20 ^B^
kidney *	0.38 ± 0.02 ^A^	0.38 ± 0.02 ^A^	0.36 ± 0.03 ^A^
spleen	0.39 ± 0.03 ^A^	0.36 ± 0.04 ^A^	0.36 ± 0.05 ^A^
lungs	0.57 ± 0.09 ^A^	0.52 ± 0.02 ^A^	0.50 ± 0.04 ^A^

Relative weight = organ weight/body weight × 100. * Considering one kidney. A, B—Different letters indicate a significant difference (*p* < 0.05) among different days for the same organ.

## Data Availability

The original contributions presented in this study are included in the article. Further inquiries can be directed to the corresponding author.

## Abbreviations

The following abbreviations are used in this manuscript:

PHT	Photohyperthermia
LED	Light Emitting Diode
MF	Magnetic Fluid
NIR	Near-Infrared
MHT	Magnetohyperthermia
